# Sequential and Personalized Linzagolix Therapy for Uterine Fibroids: A Rational Clinical Approach

**DOI:** 10.1177/26884844251380474

**Published:** 2025-09-16

**Authors:** Josep Perelló-Capó, Ignacio Cristóbal-García, Joaquim Calaf-Alsina

**Affiliations:** ^1^ Department of Obstetrics and Gynecology, Hospital de la Santa Creu i Sant Pau, Universitat Autònoma de Barcelona, Barcelona, Spain.; ^2^ Department of Obstetrics and Gynecology, Hospital Clínico San Carlos, Universidad Francisco de Vitoria, Madrid, Spain.

Uterine fibroids are estrogen-dependent benign tumors that affect up to 80% of women, often causing heavy menstrual bleeding, pelvic pain or pressure, urinary or gastrointestinal symptoms, infertility, and pregnancy-related complications.^1^

Linzagolix (LGX) is a once-daily oral GnRH antagonist that rapidly and dose-dependently suppresses the pituitary-gonadal axis.^2^ It is available in two doses: 100 mg and 200 mg (LGX100 and LGX200, respectively). LGX200 induces near-complete estradiol suppression (median <20 pg/mL), while partial suppression (20–60 pg/mL) is seen with 100 mg or with ABT.^3–5^ To mitigate hypoestrogenic side effects, an add-back therapy (ABT) combining 1 mg estradiol and 0.5 mg norethisterone acetate has been evaluated with both doses of LGX.^3^

The PRIMROSE-1 (USA, *n* = 574) and −2 (Europe, *n* = 535) phase III trials assessed the efficacy of LGX with and without ABT in premenopausal women with uterine fibroid-associated heavy menstrual bleeding during 52 weeks. In PRIMROSE-2, the bleeding response at 24 weeks was highest with LGX200 + ABT (93.9%), followed by LGX200 (77.7%), LGX100 + ABT (77.2%), and LGX100 (56.7%), compared to 29.4% with placebo, with a favorable safety profile.^3^ Regarding fibroid volume, significant reductions at week 24 were seen with LGX200 (49%), LGX200 + ABT (29%), and LGX100 (15%, *p* = 0.057), while placebo and LGX100 + ABT showed no meaningful change. A similar trend was observed for uterine volume.^3^ Importantly, volume reduction began as early as week 12 in PRIMROSE, and possibly earlier, while prior studies with GnRH agonists reported maximal reductions within 6–8 weeks, with no further benefit thereafter.^6,7^

Rebound growth of fibroids was noted when switching from LGX200 to LGX200+ABT after 24 weeks, though not to pretreatment size.^3^ This likely reflects a loss of maximal suppression, with ABT reducing the therapeutic estrogen suppression needed for tumor shrinkage. Tumor regression appears dependent on the fibrous/myometrial composition of the lesions.^8^

Limiting LGX200 monotherapy to 6 months is recommended due to concerns over bone mineral density loss.^9^ Beyond this period, either ABT must be added or the dose reduced to LGX100 (both options being suitable for long-term treatment). However, the current approach lacks a stepwise treatment strategy. In current practice, clinicians typically start with a fixed treatment and maintain it until objectives are achieved, without scheduled reassessment or adjustments.

We argue that the availability of four treatment options (LGX100 and LGX200, each with or without ABT) supports a flexible, personalized approach. A sequential three-step strategy enables tailored initiation, adjustment, and optimization of treatment according to each patient’s symptoms, goals, and treatment tolerance.

## Proposed Sequential Strategy

We propose a three-step approach: initiation, adjustment, and optimization. The initial treatment is chosen based on the primary symptom (bleeding, pain, or bulk-related complaints), followed by dose modulation and long-term stabilization.

### Initiation phase

The goal of the initiation phase is to achieve the fastest and most effective control of dominant symptoms through an intensive “induction” regimen tailored to the patient’s primary clinical presentation.

- For bleeding or pain control: Start with LGX200 + ABT (unless contraindicated). If tolerability issues arise, consider switching to LGX100 + ABT to maintain symptom control while improving tolerability.
- For fibroid/uterine size reduction: Begin with LGX200 alone. If bleeding is problematic or hypoestrogenic symptoms occur, ABT can be introduced. Notably, LGX200 + ABT is superior to LGX100 (with or without ABT) for both symptom control and volume reduction.
- If ABT is contraindicated: Start with LGX200 regardless of the symptom. Initial treatment with LGX100 results in a lower rate of bleeding reduction, less decrease in fibroid/uterine volume, a slower response time, a poorer quality of life, and lower likelihood of hemoglobin normalization at week 24 compared to LGX200.^3^

### Adjustment phase (after 8–12 weeks)

The adjustment phase aims to reduce treatment intensity and minimize long-term side effects once initial symptom control or volume reduction has been achieved. This period, typically at 8–12 weeks, coincides with the window of maximal fibroid response observed in clinical trials and prior GnRH agonist studies.

Regardless of the initial objective, the primary goal of this phase, the transition of all patients to the lowest effective dose—preferably LGX100 monotherapy—to maintain estradiol levels within the 20–50 pg/mL range, thereby sustaining symptom control while minimizing long-term hypoestrogenic risks. All adjustments should be guided by the patient’s clinical response and tolerance. Regular monitoring is recommended to ensure continued symptom control and to promptly identify any recurrence of symptoms or side effects. If symptom control is maintained on LGX100 monotherapy, this regimen should be continued as the preferred long-term strategy. However, if fibroid-induced symptoms recur, or if hypoestrogenic side effects worsen, clinicians should consider reintroducing ABT or temporarily increasing the dose to LGX200, with the aim of returning to LGX100 monotherapy as soon as stability is achieved. This individualized, stepwise approach allows for optimal long-term management, balancing efficacy, safety, and patient quality of life.

This period aims to reduce treatment intensity once the initial benefit is achieved, minimizing long-term side effects. This “window of opportunity” aligns with evidence from prior GnRH agonist studies showing maximal effect on fibroid volume within 6–8 weeks. Adjustments are symptom- and response-driven.

### Optimization phase

This final phase consists of ongoing evaluation of the patient’s status on the current regimen, with further adjustments based on evolving needs. Long-term treatment with GnRH antagonists is recommended for women with symptomatic uterine fibroids, as discontinuation is associated with high rates of recurrence of heavy menstrual bleeding.^10^ Options may include:

- Long-term medical maintenance: Continue the lowest effective dose (ideally LGX100) until the therapeutic goal is reached (e.g., pregnancy or menopause).
- Switching hormonal strategy: Consider alternatives such as the LNG-IUS for ongoing symptom control if appropriate.
- Definitive treatment: Surgical or minimally invasive options (e.g., radiofrequency ablation) if medical management is insufficient or not desired. The treatment objective shifts from active reduction to long-term stabilization with the lowest effective dose.

Regular assessments ensure the patient remains asymptomatic while minimizing risks (*e.g.,* bone density loss, hormonal side effects).

## Rationale and Future Directions

This proposed sequential approach reflects the dynamic nature of uterine fibroids treatment with oral GnRH antagonists. As the condition evolves under therapy, so must the treatment strategy, adjusting the balance between efficacy and tolerability.^11^ It also aligns with the principles of personalized medicine, offering patients individualized care adapted to their reproductive goals and symptom burden. Clinical decisions should incorporate patient preferences and be based on shared decision-making.^12^

Our recommendations are based on findings from pivotal LGX trials,^3,4^ previous studies on GnRH analogs,^6,7^ and the sequential treatment approach with LGX for adenomyosis proposed by Donnez et al.^13^ In this study, conducted with eight women, LGX administered at a fully suppressive dose of 200 mg for 12 weeks, followed by a partially suppressive dose of 100 mg for an additional 12 weeks, effectively reduced adenomyotic uterine volume and improved associated symptoms.

While this strategy is somewhat rational and appears to be supported by current evidence, prospective clinical studies are needed to validate the efficacy and safety of a sequential treatment model using LGX. Following these foundational studies, larger prospective trials will be needed to validate the long-term efficacy, patient satisfaction, and cost-effectiveness of this approach compared to fixed-dose regimens.

## Conclusion

A sequential, symptom-oriented strategy for LGX use in uterine fibroids could maximize therapeutic benefits while minimizing adverse effects. By guiding clinicians through initiation, adjustment, and optimization phases, this approach could promote tailored treatment over time, potentially improving long-term patient outcomes and quality of life.

## Abbreviations Used

ABT: add-back therapy
GnRH: Gonadotropin-Releasing Hormone
LNG-IUS: Levonorgestrel-Releasing Intrauterine System
LGX: Linzagolix
LGX100: Linzagolix 100mg
LGX200: Linzagolix 200mg
mg: milligram
n: number
pg/mL: picograms per milliliter
